# “Substance Use Disoder and Suicide Attempts”

**DOI:** 10.1192/j.eurpsy.2026.10808

**Published:** 2026-07-31

**Authors:** K. Taleb, H. Ballouk, F. Laajili, Z. Bencharfa, F. El Omari

**Affiliations:** 1 Psychiatry; 2 Arrazi Hospital, Salé, Morocco

## Abstract

**Introduction:**

Substance Use Disorders (SUDs) are recognized as a major risk factor for suicidal behavior. Frequent psychiatric comorbidities, social disintegration, and the loss of control associated with dependence further increase this vulnerability. The coexistence of addictive behaviors and mental disorders constitutes a significant public health issue, as it amplifies the risk of suicidal ideation, attempts, and completed suicide. This study aims to explore the prevalence and clinical characteristics of suicide attempts among psychiatric inpatients diagnosed with SUDs.

**Objectives:**

To assess the prevalence of suicide attempts among psychiatric outpatients with SUDs and identify associated factors.

**Methods:**

A retrospective descriptive study based on clinical records of outpatients consulting for SUDs. Data included sociodemographic profile, substances used, psychiatric comorbidities, and history of suicide attempts. Analyses were descriptive (frequencies and percentages).

**Results:**

Among 102 patients included in the study, **27%** had a documented history of suicide attempt. The majority of attempters were **men (82%)**, aged **20 to 40 years**, often presenting with significant psychosocial difficulties such as unemployment, social isolation, or family disruption.

The **main substances involved** were cannabis (70%), alcohol (55%), and polysubstance use (48%).

**Comorbid psychiatric disorders** were highly prevalent: major depressive disorder (41%), psychotic disorders (33%), and personality disorders (14%).

**Aggravating factors** included relapse history, poor adherence to treatment, and lack of social support, which significantly increased suicidal risk.

**Conclusions:**

Suicidal behavior is common among patients with SUDs, especially when comorbid depression or polysubstance use is present.

Integrated care involving psychiatric assessment, suicide risk evaluation, and addiction management is crucial to prevent recurrence and reduce mortality.

**Disclosure of Interest:**

None Declared

